# Substituting device-measured sedentary time with alternative 24-hour movement behaviours: compositional associations with adiposity and cardiometabolic risk in the ORISCAV-LUX 2 study

**DOI:** 10.1186/s13098-023-01040-x

**Published:** 2023-04-04

**Authors:** Paul J. Collings, Anne Backes, Gloria A. Aguayo, Guy Fagherazzi, Laurent Malisoux, the ORISCAV-LUX study group

**Affiliations:** 1 Physical Activity, Sport and Health Research Group, Department of Precision Health, Luxembourg Institute of Health, 1 A-B rue Thomas Edison, L-1445 Strassen, Luxembourg; 2 Deep Digital Phenotyping Research Unit, Department of Precision Health, Luxembourg Institute of Health, L-1445 Strassen, Luxembourg

**Keywords:** Inactivity, Metabolic syndrome, Obesity, Physical activity, Sleep

## Abstract

**Background:**

There is a considerable burden of sedentary time in European adults. We aimed to quantify the differences in adiposity and cardiometabolic health associated with theoretically exchanging sedentary time for alternative 24 h movement behaviours.

**Methods:**

This observational cross-sectional study included Luxembourg residents aged 18–79 years who each provided  ≥ 4 valid days of triaxial accelerometry (*n* = 1046). Covariable adjusted compositional isotemporal substitution models were used to examine if statistically replacing device-measured sedentary time with more time in the sleep period, light physical activity (PA), or moderate-to-vigorous PA (MVPA) was associated with adiposity and cardiometabolic health markers. We further investigated the cardiometabolic properties of replacing sedentary time which was accumulated in prolonged (≥ 30 min) with non-prolonged (< 30 min) bouts.

**Results:**

Replacing sedentary time with MVPA was favourably associated with adiposity, high-density lipoprotein cholesterol, fasting glucose, insulin, and clustered cardiometabolic risk. Substituting sedentary time with light PA was associated with lower total body fat, fasting insulin, and was the only time-exchange to predict lower triglycerides and a lower apolipoprotein B/A1 ratio. Exchanging sedentary time with more time in the sleep period was associated with lower fasting insulin, and with lower adiposity in short sleepers. There was no significant evidence that replacing prolonged with non-prolonged sedentary time was related to outcomes.

**Conclusions:**

Artificial time-use substitutions indicate that replacing sedentary time with MVPA is beneficially associated with the widest range of cardiometabolic risk factors. Light PA confers some additional and unique metabolic benefit. Extending sleep, by substituting sedentary time with more time in the sleep period, may lower obesity risk in short sleepers.

**Supplementary Information:**

The online version contains supplementary material available at 10.1186/s13098-023-01040-x.

## Introduction

High levels of daily sedentary time are prevalent in European adults [1, 2]. New guidelines provided by the World Health Organization (WHO) recommend that sedentary time should be limited, and highlight that replacing sedentary time with physical activity (PA) of any intensity is preferable [3]. This terminology is consistent with the evolution of an integrated movement behaviour paradigm, which acknowledges that daily time is fixed to 24 h and is subject to competing demands. That is, a change of time in one movement behaviour necessitates an equal and opposite time displacement in at least one other behaviour [4]. Compositional and non-compositional isotemporal substitution methods have been developed to accommodate theoretical switching between movement behaviours [5, 6]. They are capable of quantifying, for example, the effects upon health of substituting a fixed amount of sedentary time with alternative movement behaviours. The existing literature indicates that reallocating sedentary time to light PA or moderate-to-vigorous PA (MVPA) appears to be beneficially associated with adiposity and cardiometabolic biomarkers, with the most consistent and favourable associations attributed to MVPA [7–9]. However, a relatively limited number of studies have applied the recommended compositional method, which is specifically designed to overcome the challenge of multicollinearity between movement behaviours [10]. There is also a limited body of work in non-clinical and representative population-based samples of adults [8, 9]. This is noteworthy because the importance of PA intensity appears to differ by health status [11]. Furthermore, a number of research priorities were identified by the expert panel which assisted development of the latest WHO guidelines on PA and sedentariness. Additional research was recommended to investigate the benefits of breaking up sedentary time with light PA, and to quantify the cardiometabolic properties of interrupting prolonged sedentary time [12]. Extended periods of uninterrupted sedentariness are prevalent [1, 2] and may be particularly deleterious to cardiometabolic health [13].

The majority of European adults fail to sleep sufficiently [14] which predisposes to multimorbidity [15]. Incorporating device-measured sleep as part of 24 h time-use compositions could therefore yield findings that are highly relevant to public health. It is a weakness that most time-substitution studies have focussed on waking behaviours only, or have resorted to merging device-measured movements with questionnaire-based sleep duration [7, 8]. Time-substitution models rely on precise measurement of each movement behaviour, and self-reported sleep is systematically over-reported [16]. Furthermore, short and long sleep durations are adversely associated with health outcomes in adults [17]. Thus it is conceivable that the impact of replacing sedentary time with sleep may be contingent upon an initial sleep length. Ascertaining this will help to clarify if interventions that target sleep might have differential effects in certain sub-populations (e.g. short versus longer sleepers) and will aid the development of more specific public health recommendations and targeted interventions.

Our objective was to investigate the cross-sectional associations of device-measured 24 h time-use compositions with continuous indices of adiposity and cardiometabolic risk in a sample of Luxembourg adults from the general population. The compositional isotemporal substitution model was used to examine how statistically replacing sedentary time with the sleep period, light PA, and MVPA was associated with outcomes. We further investigated the cardiometabolic properties of artificially substituting longer with shorter bouts of sedentary time.

## Methods

### Study population

Data were from the ORISCAV-LUX 2 study, a national cross-sectional survey of cardiovascular risk factors in the Luxembourgish general adult population [18]. In total, 1558 participants were enrolled to the study in 2016-18, and detailed information about demographic, economic, lifestyle, medical and health factors were collected. Approximately one-fifth of participants did not consent to wearing an accelerometer (*n* = 345). Excluding participants with insufficient accelerometer data (*n* = 76), missing covariable information (*n* = 38; mainly dietary data and education level were missing), or missing outcome data (*n* = 53; blood biomarkers were predominantly missing), meant that 1046 participants remained for this complete-case analysis (67.1% of the starting sample). Study approval was granted by the National Research Ethics Committee (N° 201.505/12) and all participants provided written informed consent.

### Movement behaviours

An Actigraph GT3X + accelerometer (Florida, USA) was continuously worn on the wrist of the non-dominant hand (except when showering and during water activities) for one week. Data were sampled at a frequency of 30 Hz, and after download via the Actilife software (v6.13.3, Florida, USA) and calibration with the open-source R package *GGIR* (version 2.2-0), raw acceleration signals were averaged over 5 s epochs [19]. All days with  ≥ 10 h of data were considered useable and a valid wear period comprised  ≥ 4 valid days of accelerometry, including  ≥ 1 weekend day. The total sleep period was calculated as the difference between sleep onset and waking times, detected using a certified method [20]. Validated thresholds were used to estimate the average daily time that participants spent sedentary (≤ 44.8 mg), in light PA (> 44.8 to  ≤ 100.6 mg) and MVPA (> 100.6 mg) [21, 22]. We further calculated the duration of total sedentary time that was accumulated in prolonged (≥ 30 min) and non-prolonged (< 30 min) bout lengths. Full details of the objective monitoring procedure have been provided previously [23].

### Adiposity markers

Waist circumference (cm) was measured during mid-expiration with a non-distensible tape placed halfway between the lowest rib and the uppermost lateral border of the iliac crest. Total body fat (%) was assessed using the Tanita BC-418 body composition analyser (Tokyo, Japan). The instrument has been shown to perform well against dual-energy X-ray absorptiometry in healthy individuals [24].

### Cardiometabolic health biomarkers

Systolic and diastolic blood pressures (mmHg) were obtained using the automated Omron MX3 Plus blood pressure monitor (Matsusaka, Japan) after participants had been seated quietly for at least 30 min [25]. Venous blood samples were collected after overnight fasting. Biochemical analyses were performed to ascertain concentrations (mg/dL) of blood biomarkers, including fasting plasma glucose, triglycerides, high-density lipoprotein cholesterol (HDL-c), apolipoprotein A1 and B. We calculated the ratio of the two apolipoproteins. Fasting insulin (μIU/mL) was measured using an Abbott immunology analyser (chemiluminescence technique).

### Metabolic syndrome and clustered metabolic risk

For description, participants were classified as having metabolic syndrome, defined using standard criteria and suitable waist circumference thresholds (≥ 94 cm in men; ≥ 80 cm in women) [26]. For the main analysis a continuously-distributed clustered metabolic risk score was derived. The score was calculated as the sum of all *z*-scores for each component of the metabolic syndrome (waist circumference + blood pressure + fasting glucose + triglycerides + inverse HDL-c). Values for HDL-c were inverted because low values are unfavourable. The data for triglycerides were natural log transformed (to normality) prior to calculation and the *z*-score for blood pressure was taken as the arithmetic mean of systolic and diastolic values. Previous studies have constructed similar clustered risk scores [27–29]. Higher scores indicate higher cardiometabolic risk.

### Covariables

In response to a questionnaire, participants self-reported their sex (male/female), date of birth (used to calculate age), highest academic qualification (higher education/high school/no diploma), and smoking history (never/former/current smoker). The general health subscale of the short-form 36 health survey was used to indicate self-perceived general health (scores range from 0 to 100 with higher values signifying better health) [30]. Participants reported (yes/no) if they were taking any medications for hypertension, diabetes or dyslipidemia, and if they had a family history of cardiovascular disease or diabetes. A comprehensive food frequency questionnaire was used to assess dietary intake over the preceding three months [31]. We considered total caloric intake (kcal/day), salt (g/day), and caffeine consumption (g/day) as potentially important covariables. Symptoms of depression experienced in the last week were ascertained using the Center for Epidemiologic Studies Depression questionnaire (scores range from 0 to 60 with higher values indicating worse symptomatology) [32]. Sleep quality in the past month was evaluated with the validated and widely-used Pittsburgh sleep quality index (scores range from 0 to 21 with higher values representing poorer sleep quality) [33]. Time-stamped information from accelerometers was used to denote the meteorological season of measurement (summer (starting June 1st)/autumn/winter/spring).

### Statistical analysis

#### Descriptive statistics

Participant characteristics were summarised for the total sample and stratified by metabolic syndrome status. To generate 24 h movement behaviour compositions, the geometric means of each contributing behaviour were rescaled, so that all parts collectively summed to 1440 min. This was performed for two sets of compositions: (1) a four-part composition comprising the sleep period, sedentary time, light PA and MVPA, and (2) a five-part composition in which sedentary time was segregated into prolonged and non-prolonged bouts. Univariate statistics are inadequate to describe the multivariate dispersion of compositional data. Instead, pairwise log-ratios (e.g., ln [sedentary time/MVPA]) were used to convey variation between two respective compositional parts. Values closer to zero indicate higher codependency between two parts of a composition [34].

#### Time-use associations with adiposity and cardiometabolic risk markers

The compositional isotemporal substitution model was performed in accordance with available guidance [6]. The four- and five-part movement compositions were mapped into real space using isometric log-ratio transformations. Associations between compositions with each of the outcomes (modelled separately) were then assessed using linear regression models adjusted for sex, age, education level, season of measurement, smoking status, general health, medication use, and family history of cardiovascular disease or diabetes. If either of the movement compositions as a whole were significantly associated with outcomes (as determined by Wald tests), compositional isotemporal substitutions were performed to quantify the anticipated differences in outcomes associated with reallocating a fixed amount of sedentary time to one alternative behaviour (one-to-one reallocations), whilst keeping all other movement behaviours constant. The main results are expressed per 30 min time exchanges. Compositional isotemporal substitution models were initially adjusted for each of the aforementioned covariables (model A). In a more elaborate model we further adjusted for what may be perceived as either potential confounding or mediating factors, including dietary components (total caloric intake, salt and caffeine consumption), depressive symptoms, and sleep quality (model B). To examine if associations with biomarkers were independent of adiposity, in a final model, all biomarkers were further adjusted for waist circumference (model C). The data for triglycerides and fasting insulin were natural log transformed (to normality) prior to analyses. To enhance interpretability, their data have been retransformed to represent the percentage difference in outcomes per 30 min of time exchanged.

#### Time-use associations stratified by sleep period tertiles

For outcomes that exhibited significant associations with sleep period time exchanges, the data were separated by sleep period tertiles, and stratified analyses were performed to investigate heterogeneity of time-use substitutions by sleep length. For this analysis, participants were classified as either short sleepers (*n* = 349; mean ± sd sleep period: 6.6 ± 0.6 h/night), average sleepers (*n* = 349; 7.7 ± 0.2 h/night), or longer sleepers (*n* = 348; 8.8 ± 0.6 h/night). Almost all short sleepers (99.4%) failed to meet the minimum recommended sleep duration of  ≥ 7 h per night [35].

#### Sensitivity analyses

Three sensitivity analyses were performed to determine the robustness of results. First, because there is uncertainty regarding the health benefits of occupational PA, we excluded participants with manual and physically demanding jobs (*n* = 75) [36]. We further excluded shift workers (an additional *n* = 76) as they may have worked overnight and slept in the day. Second, instead of the sleep period (the time elapsed between sleep onset and final waking, inclusive of momentary waking intervals overnight), we included device-measured sleep duration in the four-part time-use composition. This necessitated redistribution of all minutes of overnight wakefulness after sleep onset [median (interquartile range): 50.4 (36.9 to 67.9) min/d) between sedentary time (47.2 (33.9 to 64.1) min/d), light PA (2.3 (1.5 to 3.3) min/d)] and MVPA (0.8 (0.4 to 1.2) min/d). Third, we specified the four-part composition based upon self-reported sleep duration. To enable this, participants reported how many hours and minutes of sleep they had achieved nightly in the past month (mean ± sd: 412.2 ± 65.6 min/d). The questionnaire emphasised that this number may be different from the number of hours spent in bed. It was not possible to recreate the 5-part time-use composition based upon either device or self-reported sleep duration, because we did not possess information about the length of sedentary bouts accrued during waking periods overnight.

#### Statistical packages

All analyses were performed using R version 4.2.1 (R Development Core Team, Vienna, Austria) using the ‘compositions’ and ‘deltacomp’ packages [37, 38]. The threshold for statistical significance was set at *p* < 0.05, but we focus our reporting and interpretation of results on 95% confidence intervals, because they indicate the range of plausible values of associations [39].

## Results

### Participant characteristics

Overall, 6263 valid days of accelerometry were recorded, with nearly all participants (98.1%) contributing four valid weekdays and an entire weekend of data. The median monitoring time was 1440 (interquartile range: 1435 to 1440) min/d. Table 1 provides a description of study participants altogether and stratified by metabolic syndrome status. Table 2 summarises the arithmetic and geometric means of movement behaviour compositions. More than half of each day was engaged in sedentary time and nearly one-third of daily time comprised prolonged sedentariness. Table 3 shows that total and prolonged sedentary time were most codependent with the sleep period. Non-prolonged sedentary time was most codependent with light PA.

**Table 1:** Participant characteristics overall and stratified by metabolic syndrome status

	All (*n* = 1046)	No metabolic syndrome (*n* = 768)	Metabolic syndrome (*n* = 278)	*p*-value
Sex				
Male	486 (46.5)	**322 (41.9)**	**164 (59.0)**	
Female	560 (53.5)	**446 (58.1)**	**114 (41.0)**	** < 0.001**
Age (y)	51.2 ± 12.2	**48.7 ± 11.7**	**58.1 ± 10.8**	** < 0.001**
Education level				
Higher education	456 (43.6)	**371 (48.3)**	**85 (30.6)**	
High school	443 (42.3)	**315 (41.0)**	**128 (46.0)**	
No diploma	147 (14.1)	**82 (10.7)**	**65 (23.4)**	** < 0.001**
Smoking status				
Never	626 (59.8)	**481 (62.6)**	**145 (52.2)**	
Former	300 (28.7)	**208 (27.1)**	**92 (33.1)**	
Current	120 (11.5)	**79 (10.3)**	**41 (14.7)**	**0.007**
Use of antihypertensive, diabetes and/or lipid-lowering medication				
No	747 (71.4)	**655 (85.3)**	**92 (33.1)**	
Yes	299 (28.6)	**113 (14.7)**	**186 (66.9)**	** < 0.001**
Family history of CVD or diabetes				
No	687 (65.7)	**523 (68.1)**	**164 (59.0)**	
Yes	314 (30.0)	**218 (28.4)**	**96 (34.5)**	
Unknown	45 (4.3)	**27 (3.5)**	**18 (6.5)**	**0.010**
Energy intake (kcal/day)	2362 (1848 to 2974)	**2284 (1806 to 2901)**	**2557 (1996 to 3136)**	** < 0.001**
Salt (g/day)	8.2 (6.2 to 11.0)	**8.1 (6.0 to 10.7)**	**8.9 (6.9 to 11.8)**	** < 0.001**
Caffeine (mg/day)	308.1 ± 313.6	298.6 ± 307.7	334.2 ± 328.5	0.06
Depressive symptoms	8 (4 to 14)	8 (4 to 14)	9 (4 to 15)	0.085
Sleep quality	5 (4 to 8)	5 (4 to 8)	5 (4 to 8)	0.45
General health	78 (70 to 86)	**78 (70 to 86)**	**74 (62 to 82)**	** < 0.001**
Season				
summer	237 (22.7)	176 (22.9)	61 (21.9)	
autumn	278 (26.6)	202 (26.3)	76 (27.3)	
winter	282 (26.9)	209 (27.2)	73 (26.3)	
spring	249 (23.8)	181 (23.6)	68 (24.5)	0.96
Waist circumference (cm)	89.8 ± 13.4	**85.7 ± 11.3**	**101.2 ± 12.0**	** < 0.001**
Systolic blood pressure (mmHg)	124.1 ± 16.8	**120.5 ± 16.3**	**134.1 ± 13.9**	** < 0.001**
Diastolic blood pressure (mmHg)	78.4 ± 10.8	**76.3 ± 10.1**	**84.1 ± 10.4**	** < 0.001**
Fasting glucose (mg/dL)	92.5 ± 13.2	**88.6 ± 8.3**	**103.4 ± 17.6**	** < 0.001**
Triglycerides (mg/dL)	88 (65 to 118)	**80 (60 to 104)**	**123 (89 to 168)**	** < 0.001**
HDL-c (mg/dL)	57.8 ± 14.2	**60.9 ± 13.5**	**49.1 ± 12.5**	** < 0.001**
CCMR (*z*-score)	0.00 ± 3.4	**− 1.3 ± 2.7**	**3.6 ± 2.8**	** < 0.001**
Total body fat (%)	28.3 ± 8.6	**26.9 ± 8.5**	**32.1 ± 7.8**	** < 0.001**
Fasting insulin (μIU/mL)	6.9 (5.1 to 9.6)	**6.2 (4.7 to 8.0)**	**10.9 (7.6 to 14.9)**	** < 0.001**
ApoB/A1 ratio	0.58 ± 0.17	**0.56 ± 0.16**	**0.65 ± 0.19**	** < 0.001**

Data are *n* (%) for categorical variables, mean ± standard deviation for normally distributed variables, and median (IQR) for non-normally distributed variables. *P*-values represent a test of group differences by metabolic syndrome status performed using chi-square tests, ANOVA or Kruskal–Wallis tests as appropriate. Bold font signifies statistically significant group differences (*p* < 0.05). Data for total body fat was available for *n* = 1025 participants (no metabolic syndrome: *n* = 751; metabolic syndrome: *n* = 274).

*ApoB/A1* Apolipoprotein B to A1, *CCMR* Clustered cardiometabolic risk, *HDL-c* High-density lipoprotein cholesterol

**Table 2:** Arithmetic and geometric compositional means for the four- and five-part time-use compositions

	Arithmetic mean		Geometric mean	
	Min/d	%	Min/d	%
Four-part composition				
Sleep period	463.4	32.2	467.6	32.5
Sedentary time	727.9	50.5	735.6	51.1
Light PA	162.7	11.3	159.3	11.0
MVPA	86.0	6.0	77.5	5.4
Five-part composition				
Sleep period	463.4	32.2	483.6	33.6
Prolonged sedentary time	442.2	30.7	432.7	30.0
Non-prolonged sedentary time	285.7	19.8	278.9	19.4
Light PA	162.7	11.3	164.7	11.4
MVPA	86.0	6.0	80.1	5.6

Geometric means are the most appropriate measure of central tendency for compositional data. The means of each contributing behaviour were rescaled so that all parts collectively summed to 1440 min.

*PA* physical activity, *MVPA* Moderate-to-vigorous physical activity

**Table 3:** Compositional variation matrices for the four- and five-part time-use compositions

	Sleep period	Sedentary time	Prolonged sedentary time	Non-prolonged sedentary time	Light PA
Four-part composition					
Sedentary time	0.05	–			
Light PA	0.11	0.14	–	–	–
MVPA	0.30	0.37	–	–	0.18
Five-part composition					
Prolonged sedentary time	0.20	–	–		
Non-prolonged sedentary time	0.22	–	0.61	–	
Light PA	0.11	–	0.43	0.06	–
MVPA	0.30	–	0.71	0.19	0.18

Pairwise log-ratios closer to zero indicate higher codependency between two compositional parts.

*PA* physical activity, *MVPA* Moderate-to-vigorous physical activity

### Time-use associations with adiposity and cardiometabolic risk markers

The four-part composition was significantly associated with adiposity indicators (*p* < 0.001), blood biomarkers (*p* ≤ 0.006), and clustered cardiometabolic risk (*p* < 0.001), but was not associated with systolic (*p* = 0.16) or diastolic blood pressures (*p* = 0.44). Table 4 contains the time substitution results for the four-part time-use composition. Replacing sedentary time with MVPA was favourably associated with lower adiposity, fasting glucose, insulin, clustered metabolic risk, and higher HDL-c (model A). Each of the associations were attenuated marginally when further adjusted for dietary components, depressive symptoms and sleep quality (model B), and associations with biomarkers were attenuated by up to half when adjusted for waist circumference (model C). Substituting sedentary time with the sleep period was favourably associated with lower adiposity markers and fasting insulin. Magnitudes of association were approximately half compared to time substitution with MVPA, and the association with fasting insulin weakened when adjusted for waist circumference (model C). Replacing sedentary time with light PA was favourably associated with lower body fat, fasting insulin, and was the only time substitution to exhibit a relationship with lower triglycerides and a lower ratio of ApoB/A1. Table 5 shows some indication that replacing prolonged with non-prolonged sedentary time was weakly associated with lower waist circumference and total body fat, but all confidence intervals narrowly spanned zero and the associations were not statistically significant. There was limited evidence of any associations with biomarkers.

**Table 4:** Four-part compositional time-use: associations with adiposity and cardiometabolic risk markers per 30 min one-to-one sedentary time exchanges

	Sedentary time → Sleep period	Sedentary time → Light PA	Sedentary time → MVPA
Waist circumference (cm)			
Model A	**− 0.63 (− 0.96 to − 0.29)**	− 0.28 (− 0.85 to 0.30)	**− 1.09 (− 1.68 to − 0.51)**
Model B	**− 0.60 (− 0.93 to − 0.27)**	− 0.34 (− 0.91 to 0.23)	**− 1.03 (− 1.60 to − 0.45)**
Fasting glucose (mg/dL)			
Model A	− 0.24 (− 0.60 to 0.12)	0.27 (− 0.35 to 0.88)	**− 1.02 (− 1.64 to − 0.39)**
Model B	− 0.23 (− 0.59 to 0.13)	0.25 (− 0.37 to 0.87)	**− 0.99 (− 1.61 to − 0.36)**
Model C	− 0.08 (− 0.43 to 0.27)	0.34 (− 0.27 to 0.94)	**− 0.73 (− 1.34 to − 0.12)**
Triglycerides (%)			
Model A	− 0.69 (− 1.91 to 0.55)	− 2.07 (− 4.14 to 0.03)	− 1.49 (− 3.59 to 0.65)
Model B	− 0.62 (− 1.84 to 0.61)	**− 2.32 (− 4.38 to − 0.21)**	− 1.19 (− 3.30 to 0.96)
Model C	− 0.02 (− 1.21 to 1.18)	− 1.98 (− 3.97 to 0.06)	− 0.17 (− 2.23 to 1.94)
HDL-c (mg/dL)			
Model A	0.14 (− 0.22 to 0.50)	0.20 (− 0.42 to 0.82)	**1.30 (0.68 to 1.93)**
Model B	0.13 (− 0.23 to 0.49)	0.24 (− 0.38 to 0.86)	**1.24 (0.61 to 1.87)**
Model C	− 0.06 (− 0.41 to 0.28)	0.13 (− 0.47 to 0.73)	**0.91 (0.30 to 1.52)**
CCMR (*z*-score)			
Model A	− 0.07 (− 0.15 to 0.00)	− 0.09 (− 0.22 to 0.05)	**− 0.28 (− 0.41 to − 0.14)**
Model B	− 0.07 (− 0.15 to 0.01)	− 0.10 (− 0.24 to 0.03)	**− 0.25 (− 0.39 to − 0.12)**
Total body fat (%)			
Model A	**− 0.29 (− 0.48 to − 0.10)**	**− 0.42 (− 0.75 to − 0.09)**	**− 0.65 (− 0.98 to − 0.32)**
Model B	**− 0.28 (− 0.47 to − 0.09)**	**− 0.45 (− 0.77 to − 0.12)**	**− 0.63 (− 0.96 to − 0.30)**
Fasting insulin (%)			
Model A	**− 2.56 (− 3.99 to − 1.11)**	**− 3.29 (− 5.71 to − 0.81)**	**− 4.71 (− 7.12 to − 2.24)**
Model B	**− 2.47 (− 3.89 to − 1.02)**	**− 3.56 (− 5.97 to − 1.08)**	**− 4.39 (− 6.81 to − 1.90)**
Model C	− 1.15 (− 2.40 to 0.12)	**− 2.90 (− 4.92 to − 0.67)**	− 2.16 (− 4.32 to 0.04)
ApoB/A1 ratio			
Model A	− 0.00 (− 0.01 to 0.00)	**− 0.01 (− 0.02 to − 0.00)**	− 0.00 (− 0.01 to 0.00)
Model B	− 0.00 (− 0.01 to 0.00)	**− 0.01 (− 0.02 to − 0.00)**	− 0.00 (− 0.01 to 0.01)
Model C	0.00 (− 0.00 to 0.01)	**− 0.01 (− 0.02 to − 0.00)**	− 0.00 (− 0.01 to 0.01)

Model A adjusted for sex, age, education level, season of measurement, smoking status, general health, medication use, and family history of cardiovascular disease or diabetes. Model B further adjusted for dietary factors (total caloric intake, salt and caffeine consumption), depressive symptoms, and sleep quality. Model C further adjusted for waist circumference. The results are β-coefficients (95% confidence intervals) and represent the expected difference in outcomes when reallocating 30 min of sedentary time to another behavior, keeping all other movement behaviours constant at the compositional mean. Data for triglycerides and insulin were on the log scale, the results have been retransformed [(expβ-1)*100] to represent the percentage difference in outcomes per 30 min of time exchanged. Bold font indicates statistically significant associations (*p* < 0.05).

*ApoB/A1* Apolipoprotein B to A1, *CCMR* Clustered cardiometabolic risk, *HDL-c* High-density lipoprotein cholesterol, *PA* Physical activity, *MVPA* Moderate-to-vigorous physical activity

**Table 5:** Five-part compositional time-use: associations with adiposity and cardiometabolic risk markers per 30 min of prolonged sedentary time exchanged with non-prolonged sedentary time

	Prolonged → Non-prolonged sedentary time		
	Model A	Model B	Model C
Waist circumference (cm)	− 0.29 (− 0.60 to 0.02)	− 0.30 (− 0.61 to 0.01)	–
Fasting glucose (mg/dL)	0.04 (− 0.29 to 0.37)	0.03 (− 0.30 to 0.36)	0.10 (− 0.22 to 0.43)
Triglycerides (%)	− 0.45 (− 1.58 to 0.69)	− 0.50 (− 1.63 to 0.64)	− 0.19 (− 1.28 to 0.91)
HDL-c (mg/dL)	0.13 (− 0.21 to 0.46)	0.14 (− 0.19 to 0.47)	0.04 (− 0.28 to 0.36)
CCMR (*z*-score)	− 0.03 (− 0.11 to 0.04)	− 0.04 (− 0.11 to 0.03)	–
Total body fat (%)	− 0.14 (− 0.32 to 0.03)	− 0.15 (− 0.32 to 0.03)	–
Fasting insulin (%)	− 0.90 (− 2.24 to 0.46)	− 0.90 (− 2.24 to 0.46)	− 0.23 (− 1.39 to 0.95)
ApoB/A1 ratio	0.00 (− 0.00 to 0.01)	0.00 (− 0.00 to 0.01)	0.00 (− 0.00 to 0.01)

Model A adjusted for sex, age, education level, season of measurement, smoking status, general health, medication use, and family history of cardiovascular disease or diabetes. Model B further adjusted for dietary factors (total caloric intake, salt and caffeine consumption), depressive symptoms, and sleep quality. Model C further adjusted for waist circumference. The results are β-coefficients (95% confidence intervals) and represent the expected difference in outcomes when replacing 30 min of prolonged with non-prolonged sedentary time, keeping all other movement behaviours constant at the compositional mean. Data for triglycerides and insulin were on the log scale, the results have been retransformed [(expβ-1)*100]–represent the percentage difference in outcomes per 30 min of time exchanged.

*ApoB/A1* Apolipoprotein B–A1, *CCMR* Clustered cardiometabolic risk, *HDL-c* High-density lipoprotein cholesterol

### Time-use associations stratified by sleep period tertiles

**Fig. 1:**
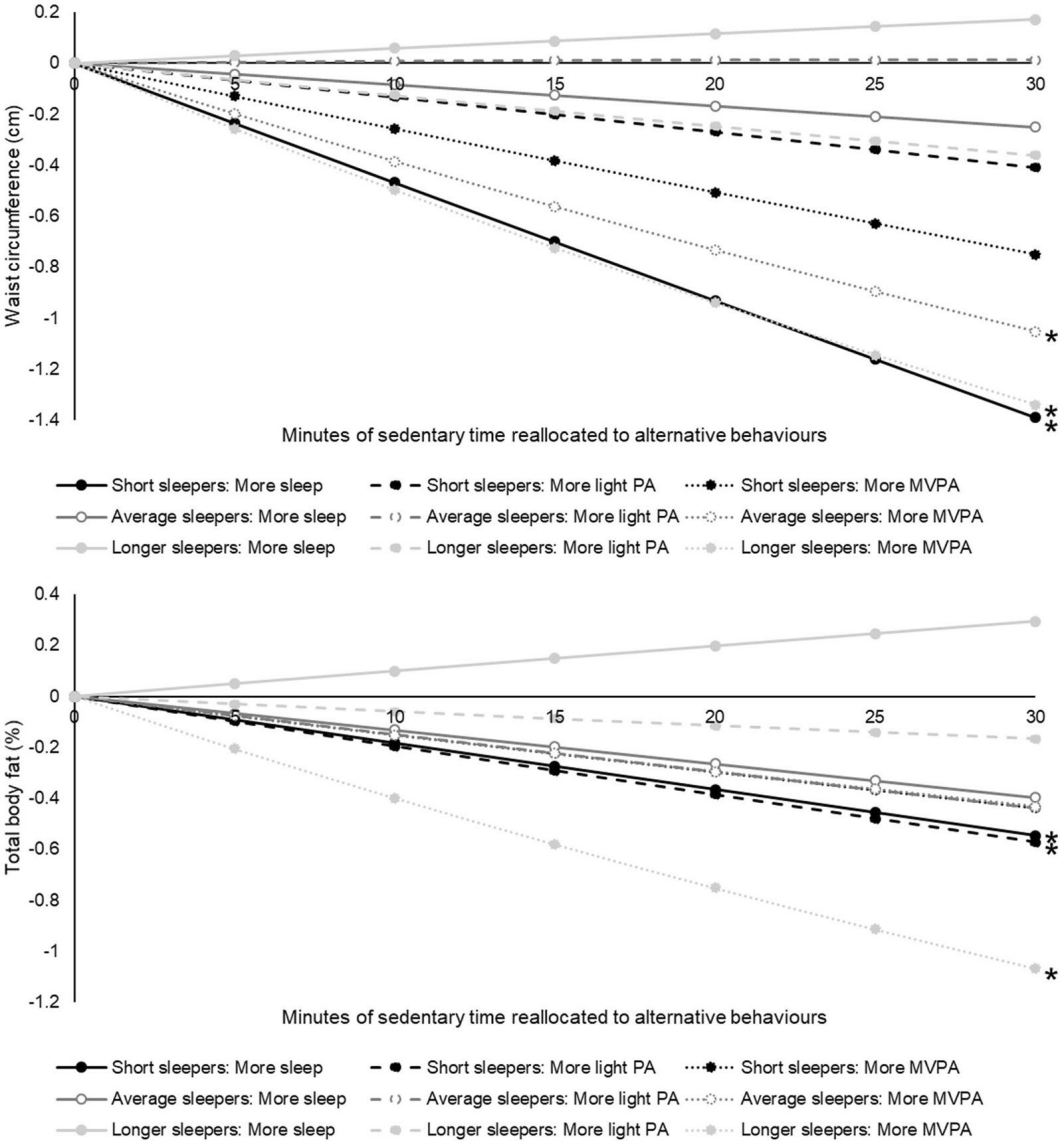
**The estimated differences in waist circumference and total body fat associated with incremental one-to-one reallocations of sedentary time to alternative movement behaviours, stratified by sleep period tertiles.** The data are β-coefficients adjusted for model A covariables (sex, age, education level, season of measurement, smoking status, general health, medication use, family history of disease) and represent the expected difference in outcomes when reallocating sedentary time to another behavior, keeping all other movement behaviours constant at the compositional mean. Asterisks indicate statistically significant associations (*p* < 0.05). Full results, including data further adjusted for model B and C covariables, are presented in Additional file 1: Table S1

Figure 1 illustrates the estimated differences in waist circumference and total body fat with incremental reallocations of sedentary time to other movement behaviours, stratified by sleep period tertiles. In short sleepers, more time in the sleep period at the expense of sedentary time was associated with lower waist circumference (β (95% CI): − 1.39 (− 2.30 to − 0.48) cm per 30 min of time exchanged) and total body fat [− 0.55 (− 1.05 to − 0.04) %]. Shifting sedentary time to light PA was also associated with lower total body fat [− 0.57 (− 1.12 to − 0.03) %] in short sleepers, but not with waist circumference [− 0.41 (− 1.39 to 0.57) cm]. Replacing sedentary time with more MVPA was associated with lower waist circumference in average sleepers [− 1.05 (− 2.10 to − 0.00) cm], and with lower values of both adiposity markers in longer sleepers (waist circumference: − 1.34 (− 2.37 to − 0.31) cm; total body fat: [− 1.07 (− 1.71 to − 0.43) %)]. There were no associations with fasting insulin across any of the sleep period strata (Additional file 1: Table S1).

### Sensitivity analyses

There was broad overlap of confidence intervals and all patterns of results were essentially unchanged (Additional file 1: Tables S2–S5). Of note, reallocating sedentary time to more self-reported sleep was not statistically significantly associated with waist circumference [β (95% CI) − 0.25 (− 0.61 to 0.11)] cm per 30 min of time exchanged or total body fat [− 0.17 (− 0.37 to 0.04) %], and point estimates were nearly half compared to time-substitutions with device-measured sleep.

## Discussion

### Summary of findings

We investigated the cross-sectional associations of replacing device-measured sedentary time with alternative 24 h movement behaviours on adiposity levels and cardiometabolic risk markers in a population-based sample of European adults. Compositional isotemporal substitution models revealed that replacing sedentary time with MVPA was beneficially associated with the widest range of outcomes. Substituting sedentary time with light PA conferred some additional and unique cardiometabolic benefit. It was the only time exchange to predict lower triglycerides and a lower ratio of ApoB/A1. Replacing sedentary time with more time in the sleep period was associated with lower fasting insulin, and extending sleep from a short to healthier length (closer to the recommended  ≥ 7 h per night) predicted lower adiposity. There was limited cardiometabolic benefit of interrupting prolonged sedentary time with shorter bouts. Our findings can be used to refine and shape plans for integrated 24 h movement behaviour guidelines, and to inform individualised and tailored interventions as part of precision medicine.

### Substitution of sedentary time with MVPA

The four-part compositional model indicated that artificially replacing sedentary time with MVPA was beneficially associated with adiposity, levels of fasting glucose and insulin, HDL-c concentration, and overall clustered cardiometabolic risk. The health benefits of MVPA are well documented, and time-substitution studies often conclude that reallocating time into MVPA from all other behaviours is most favourable [7–9]. In line with our results, beneficial time-substitution associations with clustered metabolic risk have been reported previously [27–29]. Investigations have also indicated that replacing sedentary time only with MVPA is beneficial for HDL-c [28, 40, 41], and have similarly reported that for each 30 min of time exchanged concentrations are around 1.3 mg/dL higher [28, 40, 42]. Our estimate for fasting plasma glucose is in the middle of currently reported effect sizes [41–44] and studies have typically reported larger magnitudes of association for fasting insulin [28, 41, 43, 44]. This may be due to a combination of factors, including dissimilar population characteristics, measurement methods, and statistical techniques, although differences between compositional and non-compositional time-substitution approaches are understood to be small when using isometric log-ratio transformations and linear regression [45]. We are one of several recent studies to report that, for each 30 min of sedentary time exchanged with MVPA, waist circumference is lower by approximately 1 cm [27, 28, 42, 43]. Our results exemplify the cardiometabolic importance of abdominal adiposity, as associations with biomarkers were diminished when adjusted for waist circumference. Mendelian randomization analyses have also indicated that the favourable effects of lower sedentary behaviour and higher MVPA on cardiometabolic risk are either mediated or confounded by adiposity [46].

### Substitution of sedentary time with light PA

Theoretical replacement of sedentary time with light PA was beneficially associated with total body fat, fasting insulin concentrations, and was the only time exchange to predict lower triglycerides and a lower ratio of ApoB/A1. Experimental data have likewise shown that, irrespective of the intensity of movement, interrupting sedentary time with PA reduces circulating insulin levels [47]. Observational studies have also consistently reported that light PA is associated with insulin and triglyceride concentrations, independently of MVPA [48]. In line with the existing time-substitution literature, we found that exchanging 30 min of sedentary time with light PA predicted 2 to 3% lower fasting insulin and triglycerides [27, 41, 43], but we are the first to report an association with ApoB/A1. Considering the observational nature of our study, it is noteworthy that trial data also provide empirical support to indicate that interrupting sedentariness with light PA improves lipid metabolism (reduced triglycerides and ApoB) and insulin sensitivity, potentially more than energy-matched MVPA [49]. Frequently interrupting sedentary time with light PA is hypothesised to increase lipoprotein lipase activity, hence facilitating lipid oxidation and clearance of free fatty acids [50]. For insulin sensitivity, the total cumulative PA duration is believed to be of utmost importance [51], and light PA has the advantage of being sustainable for long periods and can be used to punctuate sedentary time repeatedly throughout the day. We did not find that replacing sedentary time with light PA was advantageous for waist circumference. This probably explains why substituting sedentary time with light PA was less consistently associated with outcomes compared to MVPA. Total body fat was the only outcome for which time substitutions involving light PA and MVPA were both statistically significant in finally adjusted models. Point estimates indicated that, in order to yield an association with total body fat that was equal to replacing 30 min of sedentary time with MVPA, a 42 min swing to light PA was needed. A previous study reported similar equivalence (replacing sedentary time with 48 min of light PA, or 30 min of MVPA, returned the same association with total body fat [42]). However, a study with arguably superior characterisation of the PA intensity time-series, indicated that a much larger shift to light PA (about 82 min) was necessary to match the adiposity benefit of reallocating 30 min of sedentary time to MVPA [43].

### Substitution of sedentary time with the sleep period

Time-substitution associations between sedentary time and sleep with cardiometabolic biomarkers are inconsistent, potentially because most studies have merged device-measured movements with questionnaire-based sleep duration [7, 8]. We found that replacing sedentary time only with device-measured (and not self-reported) sleep was associated with lower adiposity indicators. We further established that replacing sedentary time with more time in the sleep period was associated with lower fasting insulin. A very similar difference (− 2.2% fasting insulin per 30 min of time exchanged) has been reported previously [41], and trials have proven that longer experimentally modified sleep predicts lower insulin [52]. Our stratified analyses by sleep period tertiles, conducted because sleep duration exhibits a nonlinear association with health outcomes [17], revealed that replacing sedentary time with more time in the sleep period was associated with lower adiposity, but only in short sleepers. It has previously been shown that shifting sleep from an inadequate to healthier length predicts attenuated gains in adiposity over time [53], and that for insufficient sleepers, replacing sedentary time with more self-reported sleep predicts lower all-cause mortality risk [54]. Our associations were independent of self-reported total caloric intake, but there may have been residual covariable effects, because short-term trials highlight that sleep extension reduces appetite and desire for unhealthy foodstuffs [55]. We propose that increasing sleep length by redistributing sedentary time may be an appropriate strategy to reduce obesity risk in short‐sleeping individuals.

### Substitution of prolonged- with non-prolonged sedentary time

Prolonged sedentary time occupied nearly one-third of daily time. The selected threshold (≥ 30 min) is somewhat arbitrary, but has been used by other studies, which also found some indication that exchanging prolonged with non-prolonged sedentary time was weakly associated with lower adiposity markers [56–58]. We found a lack of evidence for associations with biomarkers, which suggests that shorter sedentary bouts alone do not confer substantial cardiometabolic benefit, and that reallocations of sedentary time to alternative movement behaviours may be essential. In support of this assertion, replacing prolonged with non-prolonged sedentary time has been deemed insufficient to reduce the risk of premature mortality [59]. It remains unknown if there is a minimum intensity of PA that is required to confer cardiometabolic benefit. For instance, there are mixed findings about whether replacing sedentary time with standing is advantageous [45, 58]. Standing is performed upright, but often involves minimal movement and an energy expenditure < 1.5 metabolic equivalents, which is beneath the energy cost of light PA and is more consistent with sedentariness [60]. Future studies should use postural allocation algorithms or sensors to capture standing as a discrete component of 24 h time-use. This is vital because standing is often proposed as a strategy to interrupt sedentary time, despite uncertainty regarding its health enhancing credentials.

### Implications

Our results support new PA guidelines which acknowledge that replacing sedentary time with any PA intensity (including light) is beneficial for health [3]. We suggest that light PA and MVPA should feature together in public health policies and interventions that are designed to optimise cardiometabolic health. This approach is also pragmatic, since effective interventions to reduce sedentary behaviour show a consistent displacement of sedentary time to light PA, while changes in MVPA are setting-specific and difficult to achieve [61]. Although the magnitudes of some of our associations may appear to be small, the cumulative cardiometabolic effects of replacing sedentary time with alternative behaviours could be influential, as they appear to translate to reduced incidence of cardiovascular disease [62]. For short sleepers, guidelines and interventions should focus on redistributing sedentary time into the sleep period. More than half of the current study population (56.6% based on device-measured sleep duration) slept less than the recommended minimum ≥ 7 h per night [35]. If normalising sleep is causally associated with lower adiposity, it is feasible that extending sleep in habitually short sleepers could assist a downward shift in population adiposity levels.

### Strengths and limitations

We have previously observed that ORISCAV-LUX 2 study participants are generally healthier than non-participants [18, 23], but this does not necessarily threaten the validity or generalisability of our associations. Indeed, this investigation benefitted from data collected in a comparatively large population-based sample of adults, who exhibited heterogeneity in exposures and outcomes [63]. It is a strength that accelerometry was used to capture the full breadth of 24 h movement behaviours, including sleep, and unlike studies which have failed to respect the compositional nature of time-use data, the compositional isotemporal substitution model was used [10]. It is a weakness that wrist-worn accelerometers are prone to misclassifying standing as sedentary time, and a posture allocation algorithm was not used to reduce misclassifications [64]. It could also be deemed a limitation that we investigated the sleep period (which includes brief waking intervals overnight) as opposed to sleep duration. Our rationale was that momentary periods of wakefulness whilst lying in bed trying to fall back to sleep are part of a normal sleep–wake cycle, and are not likely an intervention target for reduced sedentary time [65]. Even so, the results based on sleep duration were consistent with those conditional upon the sleep period. Upon excluding manual and shift workers from the analyses, the results remained essentially unchanged, but we caution against extrapolating our findings directly to these subgroups. We have previously shown that manual workers exhibit a distinct movement behaviour profile [2] that is epitomised by less leisure-time PA and considerable occupational PA, about which the health benefits are uncertain [36]. We suggest that more time-substitution research is needed in populations with physically demanding jobs and rotating schedules including night shifts, particularly as they are high-risk groups for metabolic disease [66]. An additional caveat is that our results might not extend to other ethnicities and locations, but they can be considered widely generalisable to white European adults (60.4% of participants were born in Luxembourg, 8.3% in France, 6.3% in Portugal, and 20.2% elsewhere in Europe). As with any observational study, residual confounding or mediating effects by imperfectly measured or unspecified covariables is possible. The results are also cross-sectional and based upon theoretical not actual time reallocations. They require replication in longitudinal and experimental studies.

## Conclusion

The most efficient behaviour change scenario appears to be replacing sedentary time with MVPA, as it was beneficially associated with the broadest range of cardiometabolic risk factors. Importantly, substituting sedentary time with light PA appears to confer additional and unique metabolic benefit. It was the only time exchange to predict lower triglycerides and a lower ratio of ApoB/A1. Elongating sleep duration, by substituting sedentary time with more time in the sleep period, could lower obesity risk in short sleepers.

## Supplementary Information

**Additional file 1: Table S1.** Four-part compositional time-use: associations with adiposity indicators per 30 minute one-to-one sedentary time exchanges, stratified by sleep period tertiles. **Table S2.** Four-part compositional time-use: associations with adiposity and cardiometabolic risk markers per 30 minute one-to-one sedentary time exchanges – results excluding manual and shift workers (*n*=895). **Table S3.** Five-part compositional time-use: associations with adiposity and cardiometabolic risk markers per 30 minutes of prolonged sedentary time replaced with non-prolonged sedentary time – results excluding manual and shift workers (*n*=895). **Table S4.** Four-part compositional time-use: associations with adiposity and cardiometabolic risk markers per 30 minute one-to-one sedentary time exchanges – results based upon device-measured sleep duration. **Table S5.** Four-part compositional time-use: associations with adiposity and cardiometabolic risk markers per 30 minute one-to-one sedentary time exchanges – results based upon self-reported sleep duration.

## Data Availability

De-identified data supporting the conclusions of this article may be available upon reasonable request, if consent is provided by all authors and the ORISCAV-LUX study group. Requests to access the data should be directed to LM.

## References

[1] Loyen A, Clarke-Cornwell AM, Anderssen SA (2017). Sedentary time and physical activity surveillance through accelerometer pooling in four European countries. Sport Med.

[2] Collings PJ, Backes A, Aguayo GA (2022). Device-measured physical activity and sedentary time in a national sample of Luxembourg residents: the ORISCAV-LUX 2 study. Int J Behav Nutr Phys Act.

[3] Bull FC, Al-Ansari SS, Biddle S (2020). World Health Organization 2020 guidelines on physical activity and sedentary behaviour. Br J Sports Med.

[4] Rosenberger ME, Fulton JE, Buman MP, Troiano RP, Grandner MA, Buchner DM, Haskell WL (2019). The 24-hour activity cycle: a new paradigm for physical activity. Med Sci Sport Exerc.

[5] Mekary RA, Willett WC, Hu FB, Ding EL (2009). Isotemporal substitution paradigm for physical activity epidemiology and weight change. Am J Epidemiol.

[6] Dumuid D, Pedišić Ž, Stanford TE, Martín-Fernández J-A, Hron K, Maher CA, Lewis LK, Olds T (2019). The compositional isotemporal substitution model: a method for estimating changes in a health outcome for reallocation of time between sleep, physical activity and sedentary behaviour. Stat Methods Med Res.

[7] Grgic J, Dumuid D, Bengoechea EG, Shrestha N, Bauman A, Olds T, Pedisic Z (2018). Health outcomes associated with reallocations of time between sleep, sedentary behaviour, and physical activity: a systematic scoping review of isotemporal substitution studies. Int J Behav Nutr Phys Act.

[8] Janssen I, Clarke AE, Carson V (2020). A systematic review of compositional data analysis studies examining associations between sleep, sedentary behaviour, and physical activity with health outcomes in adults. Appl Physiol Nutr Metab.

[9] Cavallo FR, Golden C, Pearson-Stuttard J, Falconer C, Toumazou C (2022). The association between sedentary behaviour, physical activity and type 2 diabetes markers: a systematic review of mixed analytic approaches. PLoS ONE.

[10] Migueles JH, Aadland E, Andersen LB (2022). GRANADA consensus on analytical approaches to assess associations with accelerometer-determined physical behaviours (physical activity, sedentary behaviour and sleep) in epidemiological studies. Br J Sports Med.

[11] Dawkins NP, Yates T, Edwardson CL (2022). Importance of overall activity and intensity of activity for cardiometabolic risk in those with and without a chronic disease. Med Sci Sport Exerc.

[12] DiPietro L, Al-Ansari SS, Biddle SJH (2020). Advancing the global physical activity agenda: recommendations for future research by the 2020 WHO physical activity and sedentary behavior guidelines development group. Int J Behav Nutr Phys Act.

[13] Duran AT, Romero E, Diaz KM (2022). Is sedentary behavior a novel risk factor for cardiovascular disease?. Curr Cardiol Rep.

[14] Kocevska D, Lysen TS, Dotinga A (2021). Sleep characteristics across the lifespan in 1.1 million people from the Netherlands, United Kingdom and United States: a systematic review and meta-analysis. Nat Hum Behav.

[15] Ruiz-Castell M, Makovski TT, Bocquet V, Stranges S (2019). Sleep duration and multimorbidity in Luxembourg: results from the European health examination survey in Luxembourg, 2013–2015. BMJ Open.

[16] Matthews KA, Patel SR, Pantesco EJ, Buysse DJ, Kamarck TW, Lee L, Hall MH (2018). Similarities and differences in estimates of sleep duration by polysomnography, actigraphy, diary, and self-reported habitual sleep in a community sample. Sleep Heal.

[17] Chaput J-P, Dutil C, Featherstone R (2020). Sleep duration and health in adults: an overview of systematic reviews. Appl Physiol Nutr Metab.

[18] Alkerwi A, Pastore J, Sauvageot N (2019). Challenges and benefits of integrating diverse sampling strategies in the observation of cardiovascular risk factors (ORISCAV-LUX 2) study. BMC Med Res Methodol.

[19] Migueles JH, Rowlands AV, Huber F, Sabia S, van Hees VT (2019). GGIR: a research community-driven open source r package for generating physical activity and sleep outcomes from multi-day raw accelerometer data. J Meas Phys Behav.

[20] van Hees VT, Sabia S, Jones SE (2018). Estimating sleep parameters using an accelerometer without sleep diary. Sci Rep.

[21] Hildebrand M, Van Hees VT, Hansen BH, Ekelund U (2014). Age group comparability of raw accelerometer output from wrist- and hip-worn monitors. Med Sci Sport Exerc.

[22] Hildebrand M, Hansen BH, van Hees VT, Ekelund U (2017). Evaluation of raw acceleration sedentary thresholds in children and adults. Scand J Med Sci Sports.

[23] Backes A, Aguayo GA, Collings PJ (2022). Associations between wearable-specific indicators of physical activity behaviour and insulin sensitivity and glycated haemoglobin in the general population: results from the ORISCAV-LUX 2 study. Sport Med Open.

[24] Lee M, Jebb SA, Oke J, Piernas C (2020). Reference values for skeletal muscle mass and fat mass measured by bioelectrical impedance in 390 565 UK adults. J Cachexia Sarcopenia Muscle.

[25] Coleman A, Freeman P, Steel S, Shennan A (2005). Validation of the Omron MX3 Plus oscillometric blood pressure monitoring device according to the European Society of Hypertension international protocol. Blood Press Monit.

[26] Nilsson PM, Tuomilehto J, Rydén L (2019). The metabolic syndrome—what is it and how should it be managed?. Eur J Prev Cardiol.

[27] Yates T, Edwardson CL, Henson J, Zaccardi F, Khunti K, Davies MJ (2020). Prospectively reallocating sedentary time: associations with cardiometabolic health. Med Sci Sport Exerc.

[28] Whitaker KM, Pettee Gabriel K, Buman MP (2019). Associations of accelerometer-measured sedentary time and physical activity with prospectively assessed cardiometabolic risk factors: the CARDIA study. J Am Heart Assoc.

[29] Matricciani L, Dumuid D, Paquet C (2021). Sleep and cardiometabolic health in children and adults: examining sleep as a component of the 24-h day. Sleep Med.

[30] Pequeno NPF, de Cabral NLA, Marchioni DM, Lima SCVC, de Lyra CO (2020). Quality of life assessment instruments for adults: a systematic review of population-based studies. Health Qual Life Outcomes.

[31] Sauvageot N (2013). Validation of the food frequency questionnaire used to assess the association between dietary habits and cardiovascular risk factors in the NESCAV study. J Nutr Food Sci.

[32] Vilagut G, Forero CG, Barbaglia G, Alonso J (2016). Screening for depression in the general population with the center for epidemiologic studies depression (CES-D): a systematic review with meta-analysis. PLoS ONE.

[33] Mollayeva T, Thurairajah P, Burton K, Mollayeva S, Shapiro CM, Colantonio A (2016). The Pittsburgh sleep quality index as a screening tool for sleep dysfunction in clinical and non-clinical samples: a systematic review and meta-analysis. Sleep Med Rev.

[34] Dumuid D, Pedišić Ž, Palarea-Albaladejo J, Martín-Fernández JA, Hron K, Olds T (2020). Compositional data analysis in time-use epidemiology: what, why, how. Int J Environ Res Public Health.

[35] Ramar K, Malhotra RK, Carden KA (2021). Sleep is essential to health: an American academy of sleep medicine position statement. J Clin Sleep Med.

[36] Gomez DM, Coenen P, Celis-Morales C, Mota J, Rodriguez-Artalejo F, Matthews C, Saint-Maurice PF (2022). Lifetime high occupational physical activity and total and cause-specific mortality among 320 000 adults in the NIH-AARP study: a cohort study. Occup Environ Med.

[37] van den Boogaart KG, Tolosana-Delgado R (2008). “compositions”: a unified R package to analyze compositional data. Comput Geosci.

[38] Stanford T The deltacomp package. https://github.com/tystan/deltacomp. Accessed 9 March 2022.

[39] Amrhein V, Greenland S, McShane B (2019). Scientists rise up against statistical significance. Nature.

[40] Hamer M, Stamatakis E, Steptoe A (2014). Effects of substituting sedentary time with physical activity on metabolic risk. Med Sci Sport Exerc.

[41] Buman MP, Winkler EAH, Kurka JM, Hekler EB, Baldwin CM, Owen N, Ainsworth BE, Healy GN, Gardiner PA (2014). Reallocating time to sleep, sedentary behaviors, or active behaviors: associations with cardiovascular disease risk biomarkers, NHANES 2005–2006. Am J Epidemiol.

[42] Galmes-Panades AM, Varela-Mato V, Konieczna J (2019). Isotemporal substitution of inactive time with physical activity and time in bed: cross-sectional associations with cardiometabolic health in the PREDIMED-Plus study. Int J Behav Nutr Phys Act.

[43] Farrahi V, Kangas M, Walmsley R, Niemelä M, Kiviniemi A, Puukka K, Collings PJ, Korpelainen R, Jämsä T (2020). Compositional associations of sleep and activities within the 24-h cycle with cardiometabolic health markers in adults. Med Sci Sport Exerc.

[44] Ekblom-Bak E, Ekblom Ö, Bolam KA, Ekblom B, Bergström G, Börjesson M (2016). SCAPIS pilot study: sitness, fitness and fatness—is sedentary time substitution by physical activity equally important for everyone’s markers of glucose regulation?. J Phys Act Heal.

[45] Biddle G, Edwardson C, Henson J, Davies M, Khunti K, Rowlands A, Yates T (2018). Associations of physical behaviours and behavioural reallocations with markers of metabolic health: a compositional data analysis. Int J Environ Res Public Health.

[46] Wang Z, Emmerich A, Pillon NJ (2022). Genome-wide association analyses of physical activity and sedentary behavior provide insights into underlying mechanisms and roles in disease prevention. Nat Genet.

[47] Saunders TJ, Atkinson HF, Burr J, MacEwen B, Skeaff CM, Peddie MC (2018). The acute metabolic and vascular impact of interrupting prolonged sitting: a systematic review and meta-analysis. Sport Med.

[48] Amagasa S, Machida M, Fukushima N, Kikuchi H, Takamiya T, Odagiri Y, Inoue S (2018). Is objectively measured light-intensity physical activity associated with health outcomes after adjustment for moderate-to-vigorous physical activity in adults? A systematic review. Int J Behav Nutr Phys Act.

[49] Duvivier BMFM, Bolijn JE, Koster A, Schalkwijk CG, Savelberg HHCM, Schaper NC (2018). Reducing sitting time versus adding exercise: differential effects on biomarkers of endothelial dysfunction and metabolic risk. Sci Rep.

[50] Bey L, Hamilton MT (2003). Suppression of skeletal muscle lipoprotein lipase activity during physical inactivity: a molecular reason to maintain daily low-intensity activity. J Physiol.

[51] Al-Rashed F, Alghaith A, Azim R, AlMekhled D, Thomas R, Sindhu S, Ahmad R (2020). Increasing the duration of light physical activity ameliorates insulin resistance syndrome in metabolically healthy obese adults. Cells.

[52] Kothari V, Cardona Z, Chirakalwasan N, Anothaisintawee T, Reutrakul S (2021). Sleep interventions and glucose metabolism: systematic review and meta-analysis. Sleep Med.

[53] Chaput J-P, Després J-P, Bouchard C, Tremblay A (2012). Longer sleep duration associates with lower adiposity gain in adult short sleepers. Int J Obes.

[54] Stamatakis E, Rogers K, Ding D, Berrigan D, Chau J, Hamer M, Bauman A (2015). All-cause mortality effects of replacing sedentary time with physical activity and sleeping using an isotemporal substitution model: a prospective study of 201,129 mid-aged and older adults. Int J Behav Nutr Phys Act.

[55] Henst RHP, Pienaar PR, Roden LC, Rae DE (2019). The effects of sleep extension on cardiometabolic risk factors: a systematic review. J Sleep Res.

[56] Falconer CL, Page AS, Andrews RC, Cooper AR (2015). The potential impact of displacing sedentary time in adults with type 2 diabetes. Med Sci Sport Exerc.

[57] Healy GN, Winkler EAH, Brakenridge CL, Reeves MM, Eakin EG (2015). Accelerometer-derived sedentary and physical activity time in overweight/obese adults with type 2 diabetes: cross-sectional associations with cardiometabolic biomarkers. PLoS ONE.

[58] Gupta N, Heiden M, Aadahl M, Korshøj M, Jørgensen MB, Holtermann A (2016). What is the effect on obesity indicators from replacing prolonged sedentary time with brief sedentary bouts, standing and different types of physical activity during working days? A cross-sectional accelerometer-based study among blue-collar workers. PLoS ONE.

[59] Diaz KM, Duran AT, Colabianchi N, Judd SE, Howard VJ, Hooker SP (2019). Potential effects on mortality of replacing sedentary time with short sedentary bouts or physical activity: a national cohort study. Am J Epidemiol.

[60] Kowalsky RJ, Stoner L, Faghy MA, Barone Gibbs B (2021). A call to clarify the intensity and classification of standing behavior. Int J Environ Res Public Health.

[61] Segura-Jiménez V, Biddle SJH, De Cocker K, Khan S, Gavilán-Carrera B (2022). Where does the time go? Displacement of device-measured sedentary time in effective sedentary behaviour interventions: systematic review and meta-analysis. Sport Med.

[62] Walmsley R, Chan S, Smith-Byrne K, Ramakrishnan R, Woodward M, Rahimi K, Dwyer T, Bennett D, Doherty A (2022). Reallocation of time between device-measured movement behaviours and risk of incident cardiovascular disease. Br J Sports Med.

[63] Richiardi L, Pizzi C, Pearce N (2013). Commentary: representativeness is usually not necessary and often should be avoided. Int J Epidemiol.

[64] Rowlands AV, Yates T, Olds TS, Davies M, Khunti K, Edwardson CL (2016). Sedentary sphere: wrist-worn accelerometer-brand independent posture classification. Med Sci Sport Exerc.

[65] Barone Gibbs B, Kline CE (2018). When does sedentary behavior become sleep? A proposed framework for classifying activity during sleep-wake transitions. Int J Behav Nutr Phys Act.

[66] Proper KI, van de Langenberg D, Rodenburg W, Vermeulen RCH, van der Beek AJ, van Steeg H, van Kerkhof LWM (2016). The relationship between shift work and metabolic risk factors. Am J Prev Med.

